# The Arkansas Minority Barber and Beauty Shop Health Initiative: Meeting People Where They Are

**DOI:** 10.5888/pcd17.200277

**Published:** 2020-12-03

**Authors:** Chimfumnanya Smith, Austin Porter, Joyce Biddle, Appathurai Balamurugan, Michelle R. Smith

**Affiliations:** 1 Arkansas Department of Health, Little Rock, Arkansas; 2 Fay W. Boozman College of Public Health, University of Arkansas for Medical Sciences, Little Rock, Arkansas

## Abstract

**Introduction:**

The Office of Health Equity at the Arkansas Department of Health created the Arkansas Minority Barber & Beauty Shop Health Initiative (ARBBS) to address cardiovascular disease (CVD) among racial/ethnic minority populations. The objective of this study was to describe CVD-related screening results for ARBBS participants and their knowledge of CVD-related risk factors, signs, and symptoms before and immediately after participation in a screening event.

**Methods:**

ARBBS screening events were held from February 2016 through June 2019 at barber and beauty shops in 14 counties in Arkansas. During each event, participants were screened for hypertension, high cholesterol, and diabetes; surveys on CVD-related knowledge were administered before (pretest) and after (posttest) screening. Onsite public health practitioners reviewed surveys and identified abnormal screening results. Participants with abnormal screening results were counseled and given a referral to follow up with a primary care physician, wellness center, or charitable clinic. The nurse coordinator followed up to confirm that a visit or appointment had been made and provide case-management services.

**Results:**

During the study period, 1,833 people were screened. The nurse coordinator followed up with 320 (55.7%) of 574 unique referrals. Of the 574 referrals, 418 (72.8%) were for hypertension, 156 (27.2%) for high cholesterol, and 120 (20.9%) for diabetes. The overall knowledge of risk factors and symptoms of heart attack and stroke increased significantly by 15.4 percentage points from pretest to posttest (from 76.9% to 92.3%; *P* < .001). The follow-up approach provided anecdotal information indicating that several participants discovered they had underlying medical conditions and were given medical or surgical interventions.

**Conclusion:**

Through referrals and follow-ups, ARBBS participants gained greater knowledge of chronic disease prevention and risk factors. Additionally, this program screened for and identified people at risk for CVD.

**Summary**

**What is already known on this topic?**

Chronic diseases disproportionately affect racial/ethnic minority communities. Barber and beauty shops are recognized as viable locations to promote health and screen for chronic diseases.

**What is added by this report?**

This report describes screening for chronic health conditions at a barber and beauty shop–based screening program, the effect of a health education promotion campaign, and how medical referrals and participant follow-up can be integrated into screening initiatives that are based in barber and beauty shops.

**What are the implications for public health practice?**

Public health programs that seek to target racial/ethnic minority populations should meet people where they are in the community. Community-based health education and behavior modification are effective ways to decrease rates of chronic conditions among racial/ethnic minority populations.

**MEDSCAPE CME**

In support of improving patient care, this activity has been planned and implemented by Medscape, LLC, and *Preventing Chronic Disease*. Medscape, LLC, is jointly accredited by the Accreditation Council for Continuing Medical Education (ACCME), the Accreditation Council for Pharmacy Education (ACPE), and the American Nurses Credentialing Center (ANCC), to provide continuing education for the healthcare team.

Medscape, LLC, designates this Journal-based CME activity for a maximum of 1.00 AMA PRA Category 1 Credit(s)™. Physicians should claim only the credit commensurate with the extent of their participation in the activity.

Successful completion of this CME activity, which includes participation in the evaluation component, enables the participant to earn up to 1.0 MOC points in the American Board of Internal Medicine’s (ABIM) Maintenance of Certification (MOC) program. Participants will earn MOC points equivalent to the amount of CME credits claimed for the activity. It is the CME activity provider’s responsibility to submit participant completion information to ACCME for the purpose of granting ABIM MOC credit.

Release date: December 3, 2020; Expiration date: December 3, 2021

**Learning Objectives**

Upon completion of this activity, participants will be able to:

1. Assess racial and ethnic disparities in cardiovascular health in the United States
2. Distinguish the most common cardiovascular risk factors identified in the current study
3. Evaluate follow-up among adults who received abnormal results during health screenings in the current study
4. Identify which health topic was associated with the greatest gain in patient knowledge in the current study

**EDITOR**

Ellen Taratus, MS, ELS

Editor, *Preventing Chronic Disease*

Disclosure: Ellen Taratus has disclosed no relevant financial relationships.

**CME AUTHOR**

Charles P. Vega, MD

Health Sciences Clinical Professor of Family Medicine

University of California, Irvine School of Medicine

Disclosure: Charles P. Vega, MD, has disclosed the following relevant financial relationships:

Served as an advisor or consultant for: GlaxoSmithKline

**AUTHORS**

**Chimfumnanya Smith, MPH, CHES**

Arkansas Department of Health

Little Rock, Arkansas

Disclosure: Chimfumnanya Smith, MPH, CHES, has disclosed no relevant financial relationships.

**Austin Porter III, DrPH, MPH**

Arkansas Department of Health

Fay W. Boozman College of Public Health

University of Arkansas for Medical Sciences

Little Rock, Arkansas

Disclosure: Austin Porter III, DrPH, MPH, has disclosed no relevant financial relationships.

**Joyce Biddle, MPA, MPH**

Arkansas Department of Health

Little Rock, Arkansas

Disclosure: Joyce Biddle, MPA, MPH, has disclosed no relevant financial relationships.

**Appathurai Balamurugan, MD, DrPH**

Arkansas Department of Health

Fay W. Boozman College of Public Health

University of Arkansas for Medical Sciences

Little Rock, Arkansas

Disclosure: Appathurai Balamurugan, MD, DrPH, has disclosed no relevant financial relationships.

**Michelle R. Smith, PhD, MPH**

Arkansas Department of Health

Little Rock, Arkansas

Disclosure: Michelle R. Smith, PhD, MPH, has disclosed no relevant financial relationships.

## Introduction

Cardiovascular disease (CVD), the leading cause of mortality globally, represented 31.0% of all global deaths in 2017 (1). Of 17.9 million deaths worldwide from CVD in 2017, 85.0% were attributed to myocardial infarctions and stroke (2). In the United States, heart disease and stroke are the first and fifth leading causes of death, respectively (3). In 2018, 1 in every 4 deaths was associated with heart disease, and 1 in 20 deaths was associated with stroke (4).

In the United States, disparities in CVD outcomes are exacerbated by the social constructs and inequalities that disproportionately affect racial/ethnic minority populations (5). Compared with other racial/ethnic populations, Black people have the highest risk for both heart disease and stroke (5). Black people develop CVD risk factors (eg, hypertension, obesity, diabetes) at an earlier age and have a higher CVD morbidity and mortality rate compared with their White counterparts (6,7). Despite the underreporting of data among Hispanic people, heart disease accounts for 20.1% of their deaths, which is comparable to rates among Black people, at 23.7% (6).

Black people have more than twice the incidence of stroke and are twice as likely to die of a stroke compared with their White counterparts (4,8). In the United States, stroke is the third leading cause of death among Black people, fourth among Hispanic people, and fifth among White people (6). Hispanic people are more likely than non-Hispanic White people to be unaware of their risks for CVD (9).

The trends in heart disease and stroke in Arkansas are similar to national trends: they are the first and fifth causes of death, respectively (10). Of the 50 states, Arkansas ranks third highest in heart disease deaths and seventh in stroke deaths (10). The state faces significant challenges: 35.0% of adults are obese, 32.5% are physically inactive, and 22.3% are tobacco users (11). According to the US Census Bureau, Arkansas had a population of 3,017,804 people in 2019, with White people representing 79.0% of the population, Black people representing 15.7%, and other races representing the remaining 5.3% (12). Race/ethnicity plays an important role in the prevalence of CVD in the state; Black and Hispanic people have higher rates than White people of heart disease and stroke (10,13). Also, the prevalence of hypertension is higher among Black people (46.0%) than among White people (39.0%) (14). The Arkansas Red County Life Expectancy Profile shows that Black people have a lower life expectancy than their White counterparts: 68.6 years for Black men, compared with 71.1 years for White men, and 75.8 years for Black women, compared with 76.0 years for White women (15).

Barbershops and beauty shops have historically served as places where people not only get hair services but also can openly and honestly talk about issues of importance in their community (16,17). Barbershops and beauty shops are conveniently located and are frequently visited by community patrons of all ages; these locations are important and culturally appropriate avenues for addressing health and social issues (16). Health promotion programs that target Black people, particularly Black men, have partnered with barbershop owners who are trusted members of their communities to help deliver health messages and help address health issues that disproportionately affect Black communities (18). Studies have described these partnerships, demonstrated an increase in knowledge and positive changes in health behaviors among clients, and emphasized the need for community health education–based programs to increase their outreach efforts to at-risk populations through barber and beauty shop health intervention initiatives (16,18,19).

In 2013, the Office of Health Equity, formerly known as the Office of Minority Health & Health Disparities, at the Arkansas Department of Health, created the Arkansas Minority Barber & Beauty Shop Health Initiative (ARBBS) to address CVD and its risk factors among racial/ethnic minority populations. The mission of the initiative was to increase public awareness about heart disease and stroke and empower racial/ethnic minority communities to better understand hypertension prevention and management. This initiative differed from other barbershop health promotion programs in that, in addition to outreach at barbershops, it included beauty shops and barber/beauty colleges and added a program component for the Hispanic population. The initiative also included follow-up on participants who had abnormal screening results. These participants were referred to their family physician or a charitable clinic for treatment. The primary objective of this study was to describe CVD-related screening results of ARBBS participants and knowledge of CVD-related risk factors, signs, and symptoms before and immediately after participation in a screening event.

## Methods

In March 2013, the Office of Health Equity, in partnership with the cosmetology section of the Arkansas Department of Health, contacted minority-owned barbershops, beauty shops, and barber/beauty colleges and invited them to an educational session where CVD risk factors (eg, hypertension, diabetes, obesity, tobacco use) and their effect on racial/ethnic minority communities were discussed. The ARBBS initiative was introduced to the business owners, and they were asked if they would want their shops or colleges to be screening locations. To qualify as a screening site, a business was required to meet a threshold of at least 50 clients on a given Saturday, 5 to 10 barbers or beauticians working on a given Saturday, and the capacity to host 18 to 36 volunteer team members without disrupting their flow of business. All locations that met the criteria and whose owners were willing to participate signed a form approving their businesses to serve as health screening locations. This study was approved by the institutional review board at the University of Arkansas for Medical Sciences. The study was conducted in 14 counties from February 2016 through June 2019 (the study period). 

### Volunteer recruitment and training

Medical and nonmedical volunteers were recruited from local universities and colleges, the health department, local hospitals, and nonprofit organizations in the community. Volunteers recruited included physicians, advanced nurse practitioners, registered nurses, dietitians/nutritionists, certified health education specialists, public health practitioners, nursing students, pharmacy students, medical students, public health students, Spanish interpreters, and laypeople. Recruitment emails were sent out to various listservs, and flyers were printed and distributed to colleges and organizations.

All volunteers were required to attend a mandatory 2-hour standardized training before participating in the health screenings. The training included a review of a participant survey, protocols for each volunteer role, and instructions on how to administer a survey properly. Twenty-one training sessions were conducted during the 4 years, with 1,012 total volunteers in attendance.

### Participant recruitment

Study participants were recruited from the clientele of participating beauty shops and barbershops and via bilingual (English and Spanish) radio, internet, newspaper, and television advertisements. People who agreed to participate in the health screening signed a consent form that detailed the types of screening to be performed as well as their rights to confidentiality and privacy. All participants had to be aged 18 years or older.

### Screening process

The screening consisted of 8 checkpoints: 1) registration, 2) blood pressure measurement (via sphygmomanometer), 3) blood glucose and cholesterol measurement (via finger stick), 4) education on tobacco cessation, 5) education on heart disease and stroke, 6) screening for body mass index (BMI) (height and weight were measured) and education on proper nutrition and physical activity, 7) counseling and medical referrals, and 8) posttest survey. A pretest survey was administered at checkpoints 2 through 5. Volunteers were assigned to checkpoints on the basis of their training and expertise. Health educators and health practitioners conducted the educational components on CVD (heart attack and stroke), CVD risk factors (high blood pressure, high cholesterol, diabetes, and tobacco use), and proper physical activity and nutrition habits. Participants received counseling and medical referrals at checkpoint 7 from volunteers who were either medical doctors or advanced nurse practitioners. The screening process took approximately 45 minutes to an hour to complete. 

Participants who had abnormal screening results for hypertension, diabetes, or cholesterol (Box; [20,21]) were referred to medical providers or charitable clinics for further evaluation and follow-up. Participants who had a primary care physician were referred to seek treatment from their provider. Participants who did not have a primary care physician were referred to charitable clinics or medical providers in their area, regardless of their health insurance status. Participants who had a BMI of 25.0 kg/m^2^ or higher were referred to health care providers for support with proper nutrition and physical activity.

**Box. Chronic Disease Risk Levels**

**Hypertension risk (blood pressure measurements)**

Hypotension (90 mm Hg/<60 mm Hg)

Normal (91–120 mm Hg/61–80 mm Hg)

Prehypertension (121–139 mm Hg/81–89 mm Hg)

Medium stage 1 (140–159 mm Hg/90–99 mm Hg)

High stage 2 (160–179 mm Hg/100–109 mm Hg)

Critical (≥180 mm Hg/≥110 mm Hg)

**Cholesterol levels**

Hypocholesterolemia (0–49 mg/dL)

Normal (50–200 mg/dL)

Borderline (201–239 mg/dL)

High (≥240 mg/dL)

**Diabetes risk (blood glucose levels)**

Low (0–70 mg/dL)

Normal (51–140 mg/dL)

Prediabetes (141–200 mg/dL)

Diabetes (≥201 mg/dL)

**Body mass index risk (kg/m^2^)**

Underweight (<18.5)

Normal (18.5–24.9)

Overweight (25.0–29.9)

Obese (≥30.0)

**Pretest and posttest.** A pretest questionnaire and posttest questionnaire were used to obtain data on demographic characteristics, access to care, chronic disease risk levels, knowledge of chronic diseases, and medical referral status. Trained volunteers administered a paper-and-pencil bilingual (English and Spanish) survey to each participant during checkpoints 2 through 5 (pretest) and at checkpoint 8 (posttest). Each questionnaire had a unique identification number. Survey questions were adapted from the Centers for Disease Control and Prevention’s Behavioral Risk Factor Surveillance System. The pretest questions asked about demographic characteristics (age, sex, race/ethnicity, education) and whether the participant had a personal physician and health insurance. In addition, both the pretest and posttest survey assessed knowledge of chronic disease with the following multiple-choice questions: “What should a normal blood pressure be?” Responses for “top number” were less than 200, 130, 140, greater than 150, or don’t know. Responses for “bottom number” were less than 80, 90, 100, greater than 120, or don’t know. “What is a normal total cholesterol level?” Responses were less than 200, 250, 300, 400, or don’t know. Two questions were in true–false format: 1) “The following are some symptoms of a stroke” Responses were facial droop, slurred speech, weakness in arm or leg. 2) “The following are some symptoms of a heart attack” Responses were chest pain; nausea/flu-like symptoms; neck, back, and jaw pain; shortness of breath). Finally, “What is the first thing you should do if you thought someone was having a stroke or heart attack?” Responses were “take them to the hospital,” “Tell them to call their doctor,” “Call 911,” “Call their spouse or family member,” and “don’t know.”

### Medical referrals

When a participant was referred for medical follow-up, the study team initiated a new form. This bilingual medical referral form recorded the participant’s contact information and screening results and was used to track people who were referred for follow-up medical care. The unique survey number was transferred to the medical referral form if the participant received a referral. The nurse coordinator, a staff member of the Office of Health Equity, conducted follow-up telephone calls within 30 days after the screening and every 3 months thereafter for a year. During the follow-up calls, participants were asked if they kept their medical appointments, started new medications, had a change in medication, changed their dietary habits, started exercising, or quit tobacco use (if applicable); their responses were self-reported. We tracked the number of participants who kept their medical appointments and the number of participants who agreed to return to the health screening the following year. The nurse coordinator also noted any other information that the participants provided, such as whether they had received any surgical interventions as a result of the screening intervention.

### Data analysis

Our analytic sample consisted of 1,833 participants. We used descriptive statistics to summarize data on the demographic characteristics of the participants, their access to care (health insurance and personal physician), knowledge of disease, and screening results. We used data obtained from medical referral and participant follow-up forms to examine compliance (eg, keeping physician appointments or taking hypertension medication) and management of risk factors (eg, exercise, proper diet to reduce obesity, smoking cessation) among participants who received referrals. We conducted χ^2^ tests to determine whether the education received during the screening event improved the number of correct answers on the posttest. We managed all data obtained from the health screenings and follow-up telephone calls in REDCap version 9.1.20 (Vanderbilt University). We used SAS version 9.4 (SAS Institute Inc) to conduct all analyses.

## Results

Of the 1,833 participants, most (60.9%) were female, Black (62.7%), and had some college (27.3%) or were college graduates (27.5%) (Table 1). Most (54.6%) were younger than 45, most (78.6%) had health insurance, and most (68.7%) had a personal physician.

**Table 1:** Demographic Characteristics of Adults Participating in the Arkansas Minority Barber & Beauty Shop Health Initiative (N = 1,833), Arkansas, 2016–2019

Characteristic	No. (%)
**Age, y**	
18–24	284 (15.5)
25–34	371 (20.2)
35–44	346 (18.9)
45–54	270 (14.7)
55–64	312 (17.0)
≥65	212 (11.6)
Unknown/missing	38 (2.1)
**Sex**	
Male	707 (38.6)
Female	1,116 (60.9)
Unknown/missing	10 (0.5)
**Race/ethnicity**	
White	305 (16.6)
Black	1,150 (62.7)
Hispanic	311 (17.0)
Other	40 (2.2)
Unknown/missing	27 (1.5)
**Education**	
<High school graduate	262 (14.3)
High school graduate	478 (26.1)
Some college	501 (27.3)
College graduate	504 (27.5)
Unknown/missing	88 (4.8)
**Has health insurance**	
Yes	1,440 (78.6)
No	363 (19.8)
Unknown/missing	30 (1.6)
**Has a personal physician**	
Yes	1,259 (68.7)
No	473 (25.8)
Not sure/refused	2 (0.1)
Unknown/missing	99 (5.4)

Most (69.9%) were at risk for hypertension or had hypertension: 35.1% had prehypertension, 22.1% had stage 1 hypertension, 9.4% had stage 2 hypertension, and 3.3% had critical hypertension (Table 2). Most (72.2%) participants had normal cholesterol levels, most (80.2%) had normal glucose levels, and about half (49.2%) had a BMI ≥30.0.

**Table 2:** Screening and Referral Results of Adults Participating in the Arkansas Minority Barber & Beauty Shop Health Initiative (N = 1,833), Arkansas, 2016–2019

Result	No. (%)
**Blood pressure**	
Hypotension (90 mm Hg/<60 mm Hg)	5 (0.3)
Normal (91–120 mm Hg/61–80 mm Hg)	524 (28.6)
Prehypertension (121–139 mm Hg/81–89 mm Hg)	643 (35.1)
Stage 1 hypertension (140–159 mm Hg/90–99 mm Hg)	405 (22.1)
Stage 2 hypertension (160–179 mm Hg/100–109 mm Hg)	173 (9.4)
Critical hypertension (≥180 mm Hg/≥110 mm Hg)	61 (3.3)
Unknown/missing	22 (1.2)
**Cholesterol**	
Hypocholesterolemia (0–49 mg/dL)	0
Normal (50–200 mg/dL)	1,324 (72.2)
Borderline (201–239 mg/dL)	238 (13.0)
High (≥240 mg/dL)	118 (6.4)
Unknown/missing	153 (8.3)
**Blood glucose**	
Low (0–70 mg/dL)	69 (3.8)
Normal (71–140 mg/dL)	1,470 (80.2)
Prediabetes (141–200 mg/dL)	113 (6.2)
Diabetes (≥201 mg/dL)	100 (5.5)
Unknown/missing	81 (4.4)
**Body mass index, kg/m^2^**	
Underweight (<18.5)	0
Normal (18.5-24.9)	352 (19.2)
Overweight (25.0–29.9)	497 (27.1)
Obese (≥30.0)	901 (49.2)
Unknown/missing	83 (4.5)

Of the 574 unique referrals recorded during the study period, 320 (55.7%) were successfully contacted, with 161 (28.0%) keeping their appointments with their primary care provider. Through these follow-ups, we were made aware of at least 10 instances in which a participant had received surgical interventions because of abnormal screening results. Of the 574 referrals, 418 (72.8%) were for hypertension, 156 (27.2%) for high cholesterol, and 120 (20.9%) for diabetes.

The average percentage of correct answers to the questions on normal blood pressure, normal cholesterol, and what to do first if someone were having a stroke or heart attack increased from 60.8% to 87.6% (*P* < .001) from pretest to posttest (Table 3). Among the multiple-choice questions, the largest improvement was for the question, “What is a normal total cholesterol level?” The percentage increased from 44.6% to 87.9% (*P* < .001).

**Table 3:** Knowledge Assessment Results of Adults Participating in the Arkansas Minority Barber & Beauty Shop Health Initiative (N = 1,833), Arkansas, 2016–2019

Question	Correct Answer	Pretest, % Correct	Posttest, % Correct	*P* Value^a^	Percentage-Point Difference
**Multiple Choice**					
**What should a normal blood pressure level be?**	Top number <120	55.0	86.6	<.001	31.5
**What should a normal blood pressure level be?**	Bottom number <80	50.7	77.7	<.001	27.0
**What is a normal total cholesterol level?**	<200	44.6	87.9	<.001	43.3
**If you thought someone was having a stroke or heart attack, what would be the first thing you should do?**	Call 911	92.7	98.2	<.001	5.6
**Average correct**	—	60.8	87.6	—	26.8
**True or False**					
**The following are some symptoms of a stroke**					
Facial droop	True	90.4	98.5	<.001	8.1
Slurred speech	True	90.6	98.1	<.001	7.5
Weakness in arm or leg	True	89.9	95.1	<.001	5.2
**The following are some symptoms of a heart attack**					
Chest pain	True	94.1	97.4	<.001	3.3
Nausea/flu-like symptoms	True	70.4	90.0	<.001	19.6
Neck, back, and jaw pain	True	74.6	90.0	<.001	15.5
Shortness of breath	True	93.4	95.9	<.001	2.5
**Average correct**	—	86.2	95.0	—	8.8
**All**					
**Overall average correct**	—	76.9	92.3	—	15.4

a Differences between pretest and posttest determined by χ^2^ test.

The average percentage of correct answers to the true–false questions on the symptoms of a stroke and heart attack increased by 8.8 percentage points (from 86.2% to 95.0%; *P* < .001) (Table 3). Among the true–false questions, the largest improvement was in the question on symptoms of a heart attack: 70.4% of participants on the pretest and 90% on the posttest indicated that this was true, an increase of 19.6 percentage points (*P* < .001). The overall knowledge of risk factors and symptoms of heart attack and stroke increased significantly from pretest to posttest (from 76.9% to 92.3%; *P* < .001).

## Discussion

The results of this study highlight the efforts of a screening program designed to reach a population with a disproportionate share of many chronic diseases. The literature is rich in highlighting innovative ways to reach racial/ethnic minority populations, particularly Black people, to screen for specific chronic diseases (22–24). Many of these screening activities stress the importance of meeting people where they live and work and have been held in churches, barber/beauty shops, community centers, and other nontraditional locations. The ARBBS initiative sought not only to screen for chronic diseases among a high-risk population but also to provide education and refer people who required follow-up care to a health care provider.

The initial referral and nurse coordinator follow-up were unique aspects of this screening program. The program sought to identify and refer participants with abnormal screening results to an appropriate health care provider and to follow up on treatment outcomes. Using a point of contact after the initial abnormal screening results has been shown to be effective in increasing compliance (25). A study by Rorie et al used resident housing advocates (RHAs) to follow up with residents of public housing who had abnormal screening results (25). The RHA offered to help make appointments for residents and accompany residents to their follow-up appointment; the proportion of participants who completed a follow-up appointment increased from 15.0% to 55.0% (25). Although our program attempted to contact all participants with abnormal screening results, we made contact with 55.7%, and 28.0% kept their follow-up appointments within 30 days of the screening event. Nevertheless, we received anecdotal information that at least 10 participants with abnormal screening results subsequently obtained potentially life-saving surgeries.

Barber and beauty shops have been used as avenues to promote health in the Black community (16,26). Many promotion activities were associated with an increase in health knowledge. A study conducted by Luque et al administered a health education intervention in barbershops that aimed to increase the knowledge and awareness of prostate cancer and screening in the African American community (26). These researchers found a significant increase in knowledge among clients given educational materials on prostate cancer (26). Similarly, the results of our study indicated that the knowledge of chronic diseases and risk factors among participants increased significantly after the intervention.

Modifiable risk factors such as high blood pressure, obesity, diabetes, physical inactivity, and tobacco use increase CVD disparities between non-Hispanic White people and Black and Hispanic people (7,27). About 46.8% of Black people and 47.0% of Hispanic people are obese, and both populations are more likely than non-Hispanic White people to be diagnosed with hypertension and diabetes and be physically inactive (8,28). Systematic, environmental, and structural factors also contribute to the high risk and mortality rates of CVD among Black and Hispanic people (29). Racism, poverty, and low socioeconomic status are associated with increased CVD risk and mortality rates among Black people and Hispanic people (5,30). Because of inequities worsened by the social determinants of health among many members of racial/ethnic minority populations, it is essential to provide targeted educational and health-promoting interventions to these populations.

Our initiative had several strengths, including the follow-up of participants with abnormal screening results, the inclusion of Black women and Hispanic populations, and the use of nontraditional locations. In addition, more than half of participants were aged 45 or younger; information obtained by people at these younger ages may help to reduce the risk for chronic diseases later in life. Other health promotion activities, such as screening for HIV and sexually transmitted infections, breast cancer, prostate cancer, and mental health, can easily be incorporated into the structure of our initiative. Programs such as ours can be sustained through in-kind contributions and collaboration with various partner organizations, such as hospitals, universities or colleges, and other local community organizations.

Our study has several limitations. First, we used a convenience sample. Barbershop and beauty shop clientele who self-selected to participate in the program may have been different from those who elected not to participate. Second, we did not measure the long-term effect of knowledge gained during the intervention. We assessed knowledge gained immediately after the intervention. Future studies should be designed to measure the long-term effects of the program on participants’ knowledge and changes in health outcomes. Third, we did not conduct regression analyses to identify variables such as health insurance status, ethnicity, and sex that may be associated with keeping follow-up appointments. Fourth, we did not collect data on people lost to follow-up; this information could have provided additional insight into the effect of our intervention. Future research should explore other types of follow-up interventions, such as medication therapy programs for populations with limited access to these programs. Additionally, a cost-benefit analysis of the initiative should be conducted.

Notwithstanding these limitations, the results of our study add to the evidence that barber and beauty shops are viable options for promoting healthy behaviors and conducting screening programs in racial/ethnic minority communities. To the best of our knowledge, our program was the first to incorporate Black women and Hispanic participants. Participants were screened for chronic health conditions and received education on how to reduce their risk for these conditions. Follow-up on abnormal screening results was a critical element of our program: it sought to ensure that patients were further tested and treated by a medical provider. Screening programs must be intentional in screening, educating, and intervening with populations at risk of chronic diseases. Public health programs that seek to target racial/ethnic minority populations should meet people where they are in the community. Community-based health education and behavior modification can be effective measures to decrease CVD risk factors in racial/ethnic minority populations.

## Post-Test Information

To obtain credit, you should first read the journal article. After reading the article, you should be able to answer the following, related, multiple-choice questions. To complete the questions (with a minimum 75% passing score) and earn continuing medical education (CME) credit, please go to http://www.medscape.org/journal/pcd. Credit cannot be obtained for tests completed on paper, although you may use the worksheet below to keep a record of your answers.

You must be a registered user on http://www.medscape.org. If you are not registered on http://www.medscape.org, please click on the “Register” link on the right hand side of the website.

Only one answer is correct for each question. Once you successfully answer all post-test questions, you will be able to view and/or print your certificate. For questions regarding this activity, contact the accredited provider, CME@medscape.net. For technical assistance, contact CME@medscape.net. American Medical Association’s Physician’s Recognition Award (AMA PRA) credits are accepted in the US as evidence of participation in CME activities. For further information on this award, please go to https://www.ama-assn.org. The AMA has determined that physicians not licensed in the US who participate in this CME activity are eligible for AMA PRA Category 1 Credits™. Through agreements that the AMA has made with agencies in some countries, AMA PRA credit may be acceptable as evidence of participation in CME activities. If you are not licensed in the US, please complete the questions online, print the AMA PRA CME credit certificate, and present it to your national medical association for review.

## Post-Test Questions

### Study Title: The Arkansas Minority Barber and Beauty Shop Health Initiative: Meeting People Where They Are

#### CME Questions

1. Which of the following statements regarding cardiovascular risk according to race/ethnicity is most accurate?
  - Latinx adults are at the highest risk for heart disease; Black adults are at the highest risk for stroke
  - Black adults are at the highest risk for heart disease; Latinx adults are at the highest risk for stroke
  - Latinx adults are at the highest risks for heart disease and stroke
  - Black adults are at the highest risks for heart disease and stroke
2. Which of the following illnesses was most prevalent in screening results from the current study?
  - Hyperlipidemia
  - Type 2 diabetes
  - Obesity
  - Hypertension and prehypertension
3. What were the approximate follow-up rates after an abnormal community-based screening in the current study?
  - 83% of patients contacted; 67% kept follow-up appointments with primary care
  - 68% of patients contacted; 50% kept follow-up appointments with primary care
  - 56% of patients contacted; 28% kept follow-up appointments with primary care
  - 42% of patients contacted; 13% kept follow-up appointments with primary care
4. Which of the following questions was associated with the most positive change from pre-intervention to post-intervention survey in the current study?
  - What is a normal systolic blood pressure?
  - What is a normal diastolic blood pressure?
  - What is a normal total cholesterol level?
  - What is the first thing to do in case of suspected stroke or heart attack?
